# Neural reactivity to infant faces and trait mindfulness as prospective predictors of postpartum depressive symptoms

**DOI:** 10.3758/s13415-025-01319-8

**Published:** 2025-06-20

**Authors:** Sarah E. Woronko, Emilia F. Cárdenas, Christian A. L. Bean, Resh S. Gupta, Kathryn L. Humphreys, Autumn Kujawa

**Affiliations:** 1 Department of Psychology and Human Development, Vanderbilt University, Nashville, TN USA; 2 Department of Psychological & Brain Sciences, Washington University in St. Louis, St. Louis, MO USA

**Keywords:** Depression, LPP, Pregnancy, Mindfulness

## Abstract

Postpartum depression (PPD) impacts the health of both mothers and their offspring, underscoring the importance of early identification of risk factors for PPD. While both low-trait mindfulness and blunted neural processing to emotional stimuli (indexed by the late positive potential; LPP) have been separately associated with depression, previous work has highlighted an inverse relationship between trait mindfulness and neural emotional processing. Thus, it remains unclear how facets of trait mindfulness and neural emotional processing interact as risk factors for PPD. During the second trimester, pregnant women (*n* = 117) completed the Five Facet Mindfulness Questionnaire, the Inventory of Depression and Anxiety Symptoms (IDAS), and an infant face matching task while continuous electroencephalography was recorded. At 9 weeks postpartum, participants’ PPD symptoms were reassessed with the IDAS. A series of hierarchical linear regression models revealed that acting with awareness, a trait mindfulness facet, and LPP to happy infant faces interacted to predict PPD symptoms (β = .217, *p* = .014, 95% CI [.045, .390]) after adjusting for depression levels in mid-pregnancy, such that low acting with awareness was associated with greater PPD symptoms when LPP to happy infant faces was 1 standard deviation below (β = −.548, *SE* = .150 , *p* < .001) and at the mean (β = −.309, *SE* = .106, *p* = .004). Findings suggest that an enhanced LPP to positively valenced stimuli may be protective against postpartum depression for those with low-trait mindfulness.

**Supplementary Information:**

The online version contains supplementary material available at 10.3758/s13415-025-01319-8.

## Introduction

Globally, nearly one in six women develop postpartum depression (PPD) after giving birth (Hahn-Holbrook et al., 2018), and PPD has been associated with worsened health outcomes for both mothers and offspring (Takács et al., 2020; Weissman et al., 2006). Thus, identifying early risk and resilience factors for the development of PPD is an ongoing priority. Given the known role of aberrant emotion processing and regulation in the development and maintenance of depression (Joormann & Siemer, 2011; Joormann & Stanton, 2016), both trait mindfulness (i.e., tendency to be mindful: existing in the present moment with acceptance and nonjudgment) and the late positive potential (i.e., a posterior event-related potential (ERP) following emotionally salient stimuli indexing arousal; MacNamara et al., 2022) have been separately studied in the context of depression (Carpenter et al., 2019; Proudfit et al., 2015). Conceptually, the combination of limited self-reported present-moment awareness (i.e., low-trait mindfulness) along with tendencies to disengage from social-emotion cues at the neural level (i.e., blunted LPP) could increase risk for developing depressive symptoms, particularly during times of major transition, such as pregnancy. Prior work has highlighted how mindful states may impact neural indicators of emotional processing (Egan et al., 2018), yet the ways in which both mindfulness tendencies and neural responses to emotional stimuli interact to shape depression risk have yet to be examined.

High-trait mindfulness has been shown to protect against the development of depression in both pregnant and nonpregnant samples (Carpenter et al., 2019; Godbout et al., 2023). Additionally, the practice of mindfulness-based interventions to bolster both state and trait mindfulness has been shown to effectively reduce depressive symptoms (Min et al., 2023). Importantly, trait mindfulness is a complex phenomenon with multiple features in which individuals may vary. For example, trait mindfulness includes both the act of bringing attention to one’s inner and outer experiences and stimuli (i.e., being present) in addition to the way one does so (i.e., nonjudgmentally; Van Dam et al., 2018) which may uniquely function in the promotion of mental well-being. To better understand underlying constructs of trait mindfulness, Baer and colleagues (2006) performed a factor analysis on the combined items of mindfulness questionnaires to identify the factor structure of mindfulness. Five factors emerged: 1) Nonreactivity to inner experiences, 2) Observing or attention to sensations, perceptions, thoughts, and feelings, 3) Acting with awareness, 4) Describing or labeling feelings with words, and 5) Nonjudgment of experience.

The five-factor structure was further tested in a confirmatory factor analysis, leading to the development of the Five Facet Mindfulness Questionnaire (FFMQ; Baer et al., 2006). The FFMQ is a measure of trait mindfulness and has been used to isolate the facets of trait mindfulness most salient for promoting psychological well-being. Of the five facets of mindfulness, nonreactivity, nonjudgment, and acting with awareness have been found to be the most robust predictors of general psychological distress (de Bruin et al., 2012) and of depression, more specifically (Desrosiers et al., 2013). Within perinatal periods, elevated nonjudgment and acting with awareness were found to be uniquely predictive of less difficulty bonding with one’s infant (Garon-Bissonnette et al., 2024). Additionally, women with low levels of depression throughout pregnancy and postpartum reported higher levels of acting with awareness and nonjudgment compared with women who reported elevated depressive symptoms (Hulsbosch et al., 2023), further supporting the importance of specific facets of mindfulness for understanding depression risk.

At the neural level, the late positive potential (LPP) is an indicator of arousal and allocation of cognitive resources in response to salient or emotional stimuli that has been shown to be altered in depression (Hajcak & Foti, 2020). The LPP is a sustained, positive event-related potential that begins approximately 400 ms following stimulus presentation and can be manipulated with emotion regulation strategies (i.e., reappraisal; MacNamara et al., 2022). In line with the emotion context insensitivity theory (Rottenberg et al., 2005), which posits that individuals with depression canonically show disengagement with and low reactivity to emotional stimuli, numerous studies have reported that individuals with elevated depressive symptoms show blunted LPP to emotional stimuli of both positive and negative valences (Hill et al., 2019; Weinberg et al., 2016). Importantly, pregnancy is a time of considerable neurophysiological changes (e.g., Cárdenas et al., 2019), so neural underpinnings of emotion processing and regulation, such as those captured in the LPP, may be particularly salient at this time for postpartum mental health.

Although ERP studies of depression have used a broad range of emotional images to elicit the LPP, emotional infant faces are especially relevant social-emotional stimuli during the peripartum period as parents prepare for caregiving responsibilities. Prior research has shown that adults have a heightened LPP to crying infant faces relative to neutral or positive faces (Kuzava et al., 2020). While the magnitude of LPP to emotional infant faces does not appear to differ for parents compared with adults without children, other ERPs (e.g., N170) and activation of brain regions relevant to facial and emotional processing (e.g., amygdala and insula) have been shown to be elevated in parents compared with adults without children (Bjertrup et al., 2019; Vuoriainen et al., 2022), highlighting important neurological changes associated with responsiveness to infant cues in caregiving. Additionally, neural measures of infant face processing have been shown to be impacted by depression (see Bjertrup et al., 2019, for review). For example, elevated perinatal depressive symptoms were associated with blunted LPP to distressed infant faces cross-sectionally (Rutherford et al., 2016), but another study observed a positive relationship between LPP to happy infant faces during pregnancy and postpartum depressive symptoms (Mulligan et al., 2022), underscoring the need for further research probing the relationship between LPP and PPD.

Both low-trait mindfulness and blunted LPP have been separately associated with increased depressive symptoms (Hill et al., 2019, Hulsbosch et al., 2023). Importantly, mindfulness as an emotion regulation strategy has also been used to manipulate LPP amplitude. For example, compared with passive viewing, LPP to emotional stimuli was elevated when participants adopted a mindful viewing state (Egan et al., 2018). However, another study found no difference in LPP amplitude between passive viewing and mindful states (Lin et al., 2016). In addition to studying effects of state mindfulness, some studies have shown that high trait mindfulness was related to blunted LPP amplitude to both positive and negative stimuli (Brown et al., 2013; Lin et al., 2016), but these findings were not replicated in other studies (Eddy et al., 2015; Egan et al., 2018). Thus, it remains unclear how mindfulness and LPP both interact and independently impact depression risk. This is important to examine as both low-trait mindfulness and blunted LPP are related to elevated depression (Desrosiers et al., 2013; Hill et al., 2019), but inversely related to each other (Brown et al., 2013; Lin et al., 2016). Further, as Treves and colleagues (2024) note, mindfulness has historically been treated as a univariate construct in the neurophysiological literature despite the multitude of differences in how facets of mindfulness uniquely relate to psychopathology (Carpenter et al., 2019), suggesting the need to consider individual facets of trait mindfulness rather than trait mindfulness generally in terms of interactions with emotional reactivity and associations with mental health.

The goal of the current study was to examine both facets of trait mindfulness and the LPP to contextually salient emotional cues as prospective predictors of PPD symptoms. In addition, we examined the combined effects of mindfulness and the LPP, including both the extent to which individual differences in trait mindfulness were related to LPP magnitude and whether mindfulness and LPP interact to predict PPD symptoms. Pregnant women reported facets of trait mindfulness and depressive symptoms in the second trimester. Additionally, participants completed an infant face matching task where they were presented with images of shapes and neutral, distressed, and happy infant faces that has been previously shown to elicit a reliable LPP (Cárdenas et al., 2023). At 9 weeks postpartum, participants again reported depressive symptoms to assess how perinatal mindfulness facets and LPP interacted in the prediction of PPD. Given previous work that has highlighted the importance of acting with awareness and nonjudgment facets in depression and postpartum depressive symptoms (Hulsbosch et al., 2023), we hypothesized that both subscales would predict postpartum depressive symptoms. Additionally, we hypothesized that a relatively blunted LPP to emotional infant faces relative to nonsocial stimuli (i.e., shapes) would be related to elevated postpartum depressive symptoms in line with previous work (Hill et al., 2019; Rutherford et al., 2016). Finally, given that low-trait mindfulness and blunted LPP are both associated with elevated depression, we anticipated that there would be an interaction between acting with awareness and nonjudgment subscales with LPP to emotional infant faces such that the combination of low mindfulness and blunted LPP would be associated with the highest postpartum depressive symptoms.

## Method

### Participants

The sample consisted of pregnant individuals (*N* = 120) recruited from local prenatal clinics and through social media advertisements. This sample has previously been reported on by Cárdenas and colleagues (2023). Exclusion criteria included carrying more than one fetus, being older than 40 years old, and endorsing bipolar disorder, psychosis, or borderline personality disorder. Following informed consent, three participants did not complete any portion of the study and were excluded from further analyses. The final sample (*n* = 117), all of whom identified as women, had a mean age of 30.63 years (*SD* = 4.72 years), and a racial and ethnic composition as follows: White (*n* = 93, 79.5%), Black (*n* = 12, 10.2%), Asian (*n* = 3, 2.6%), Hispanic/Latinx (*n* = 11, 9.4%), and any other race (*n* = 9, 7.7%). At baseline, 14 participants (12.0%) were taking a psychotropic medication. Additionally, parity status was as follows: primiparous (*n* = 76, 65.0%) and multiparous (*n* = 41, 35.0%).

### Procedure

All study procedures were approved by the Institutional Review Board at Vanderbilt University. After initial screening, eligible participants completed the FFMQ (Baer et al., 2006) and the general depression subscale of the Inventory for Depression and Anxiety Symptoms (IDAS; Watson et al., 2007) during the second trimester of pregnancy to assess trait mindfulness facets and depressive symptoms during pregnancy, respectively. On average, participants were 21.80 ± 3.16 weeks pregnant (range: 17–29 weeks) at the completion of Wave 1. Participants also completed an infant face matching task where they matched infant faces or shapes while continuous EEG data was collected. At nine weeks postpartum (9.22 ± 2.53 weeks postpartum; range: 6–22 weeks), participants’ depressive symptoms were reassessed with the general depression subscale of the IDAS. Participants’ birth outcomes were also assessed. On average, babies were born at 39.14 ± 1.52 weeks (range: 34.57–42.29 weeks) with a sex distribution as follows: boys (*n* = 41, 35%), girls (*n* = 49, 41.9%), and missing (*n* = 27, 23.1%). Twenty-six infants (22.2%) were in the neonatal intensive care unit following delivery.

### Measures

#### Five Facet Mindfulness Questionnaire (FFMQ; Baer et al., 2006)

The FFMQ is a 39-item self-report questionnaire assessing five dimensions of mindfulness:

1. Acting with awareness (reverse coded: e.g., “*When I do things, my mind wanders off and I’m easily distracted*”);
2. Nonreactivity to one’s inner experiences (e.g., “*I perceive my feelings and emotions without having to react to them*”);
3. Nonjudgment about one’s inner experiences (reverse coded: e.g., “*I criticize myself for having irrational or inappropriate emotions*”);
4. Observing one’s inner experiences (e.g., “*I pay attention to how my emotions affect my thoughts and behavior*”); and
5. Describing or labeling one’s emotions (e.g., “*I’m good at finding words to describe my feelings*”).

Items are rated on a scale from 1 (*never or very rarely true*) to 5 (*very often or always true*) and partitioned into the five mindfulness subscales specified above. Subscale scores are the sum of their items such that possible nonreactivity subscale scores range from 7 to 35, and the remaining subscales range from 8 to 40 with higher scores indicative of higher subscale endorsement. The FFMQ has been validated for use in pregnancy (Kantrowitz-Gordon, 2018). Internal consistency ranged from acceptable to excellent in this sample (acting with awareness α = 0.91, nonreacting α = 0.74, nonjudging α = 0.93, observing α = 0.80, and describing α = 0.93).

#### Inventory of Depression and Anxiety Symptoms (IDAS; Watson et al., 2007)

The 64-item version of the IDAS, a self-report questionnaire assessing internalizing symptoms, was administered at the initial assessment in pregnancy and again postpartum. Items are rated on a scale from 1 (*not at all*) to 5 (*extremely*). The general depression subscale used for subsequent analyses included 20 items that assess depressive symptoms. Scores on the general depression subscale range from 20 to 100 with higher scores indicative of elevated depressive symptoms. Internal consistency ranged from good to excellent in this sample (pregnancy α = 0.92 and postpartum α = 0.87). The IDAS has been extensively validated (Watson et al., 2007) and previously used to measure depressive symptoms in pregnancy (Miller et al., 2017). Of note, 23 participants (19.7%) did not complete the postpartum depressive symptom measure. Notably, most of the sample was below the IDAS clinical screening cut off for major depressive disorders (≥46.5; Stasik-O’Brien et al., 2018) during pregnancy (82.1%) and postpartum (88.9%).

#### Infant Face Matching Task (IFMT; Cárdenas et al., 2023)

**Fig. 1:**
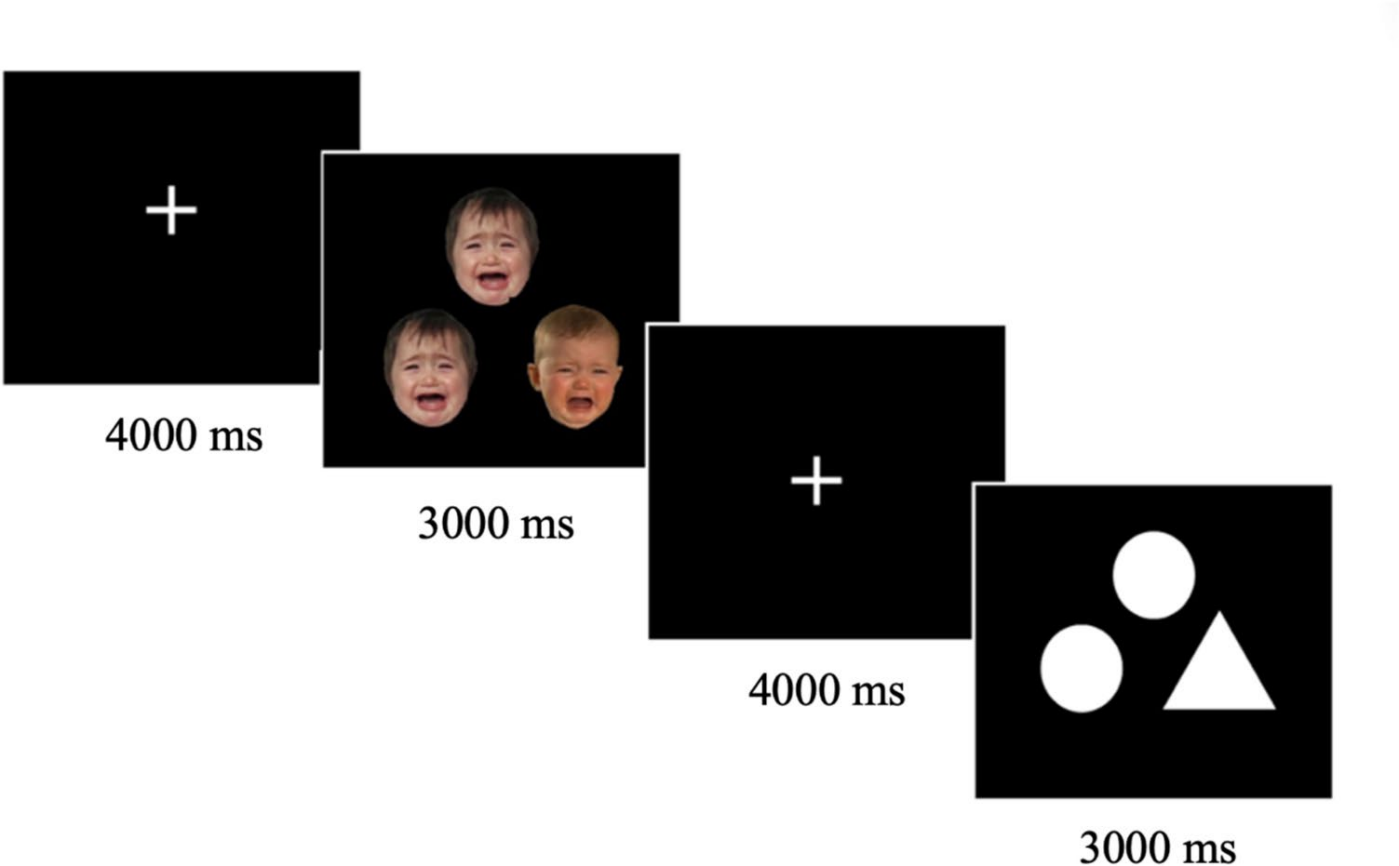
Example of a distressed trial and shape trial in the infant face matching task

During the IFMT, participants were presented with three stimuli in a triangular formation (see Fig. 1). Participants were asked to respond to the bottom stimulus that matched the center stimulus by clicking the mouse on the corresponding side. The task included 20 trials of four types: happy, distressed, and neutral infant faces, and shapes for a total of 80 trials. Each trial was presented for 3,000 ms and preceded by a 4,000 ms fixation cross. The task was split into two blocks of 40 trials, separated by a break.

### EEG Collection and Preprocessing

EEG was collected continuously using a 32-electrode BrainVision actiCAP (Munich, Germany) in a 10–20 layout while participants completed the IFMT. Four facial electrooculogram (EOG) electrodes were placed 1 cm outside of each eye and above and below the right eye to capture horizontal and vertical ocular movements. Data were collected using an actiChamp amplifier, sampled at 1000 Hz, and online referenced to Cz. During data collection, electrode impedances were maintained below 30 kΩ. EEG data from a subset of participants (*n* = 54, 47.4%) collected during the COVID-19 pandemic were down-sampled to 16 electrodes, and electrode density was included as a covariate in all regression models.

All EEG preprocessing was completed in BrainVision Analyzer (Version 2.3; Brain Products GmbH, Gilching, Germany). Data was passed through a Butterworth bandpass filter (0.01 Hz high pass cutoff, 30 Hz low-pass cutoff) and notch filter at 60 Hz. Filtered data were re-referenced to the mastoids (TP9 and TP10), segmented into 3,200 ms windows (200 ms before to 3,000 ms after stimuli presentation), and corrected for ocular artifacts using EOG electrodes with the Gratton–Coles method (Gratton et al., 1983). For EEG data collected from 16 electrode montages and data from 32 electrode montages with missing EOG electrodes, FP1 was used to correct for vertical eye movement, and FT9 and FT10 were used to correct for horizontal eye movement (Pegg et al., 2024). Additional artifacts were removed using semi-automatic artifact rejection, excluding voltage steps larger than 50 µV, 400 ms intervals with voltage ranges larger than 175 µV, voltage amplitudes greater than 200 µV or less than −200 µV, 100 ms intervals with voltage differences less than 0.5 µV, and any other artifact noted by visual inspection. Baseline correction from the 200 ms preceding stimulus presentation was applied to the 3000 ms window following stimulus presentation.

**Fig. 2:**
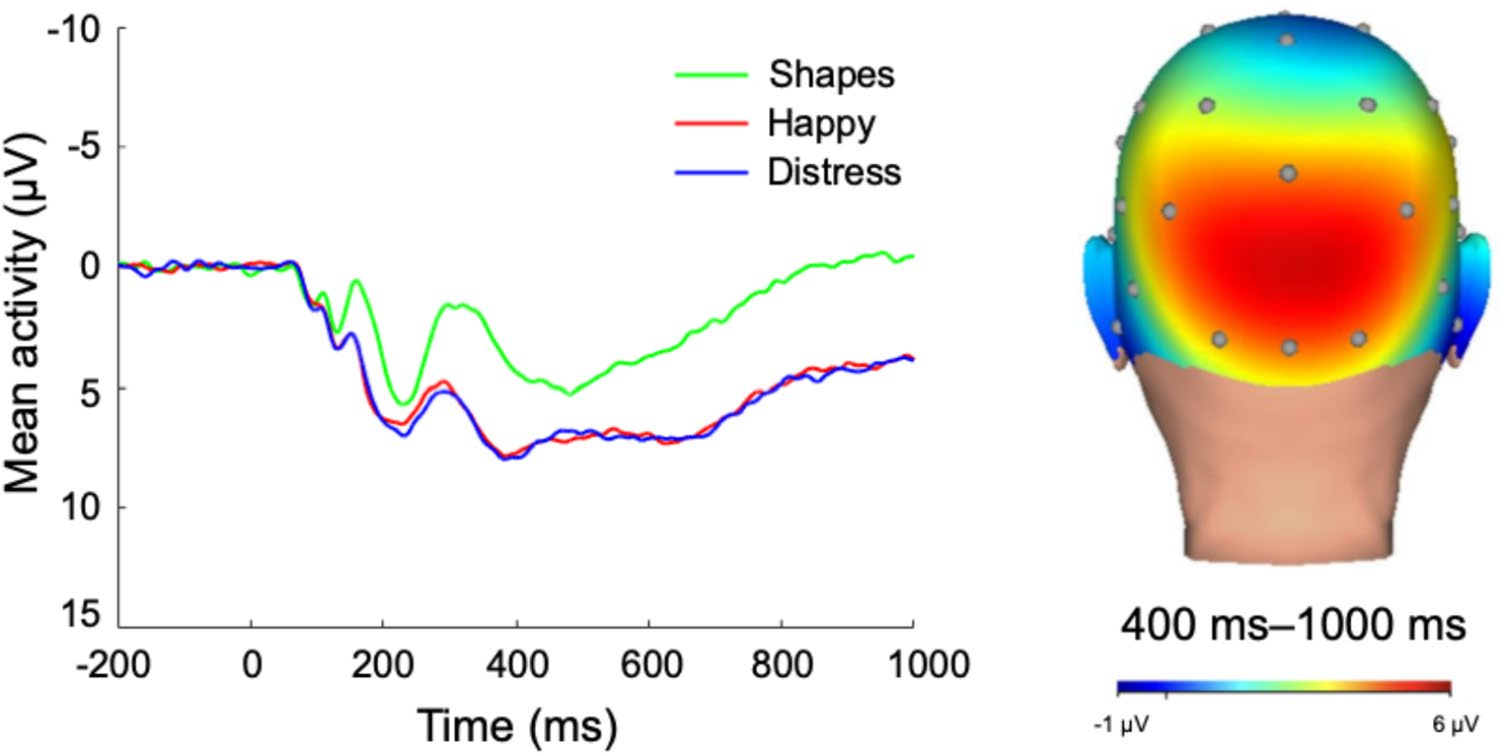
Average ERP waveforms (negative up) for shape, happy, and distressed conditions at occipitoparietal electrodes (O1, O2, Oz, Pz, CP1, CP2) 400–1,000 ms following stimulus presentation in the infant face matching task (left). The associated scalp topography (right) shows the distribution of responses to emotional vs. neutral (shape) stimuli and is associated with the late positive potential (LPP) event-related potential (ERP) 400–1,000 ms following stimulus presentation. (Color figure online)

Segmented, artifact-free data was separated by trial type (i.e., happy, distress, neutral, shapes), averaged, and pooled (CP1, CP2, O1, O2, Oz, Pz), and any incorrect trials were excluded from ERP averages (accuracy rate as follows: happy = 98.6%, distressed = 97.9%, neutral = 98.4%, and shapes = 98.3%). These electrodes were chosen based on their location over occipitoparietal regions where LPP is maximal (Foti et al., 2009) and their shared placement on both a 32- and 16-electrode cap montage. Pooled data were exported into windows 400 ms to 1,000 ms following stimulus presentation for approximation of LPP (see Fig. 2; Foti & Hajcak, 2008; Stange et al., 2017). Following preprocessing and exclusion of bad segments, 18 ± 2.6 average trials remained for distressed condition, 18 ± 2.3 trials for happy condition, 18 ± 2.3 trials for neutral condition, and 18 ± 2.7 trials for shape condition.

Unstandardized residuals were calculated for happy and distressed trials, controlling for LPP to shapes, resulting in the LPP to distressed and LPP to happy infant faces used in subsequent analyses. The shape condition was chosen as the baseline comparison because ample research highlights that neutral faces are not necessarily processed as neutral stimuli, especially for individuals with psychopathologies (Filkowski & Haas, 2017). Additionally, previous iterations of this task canonically control for response to shapes rather than neutral faces (Kujawa et al., 2015; MacNamara et al., 2018). However, given that a subset of previous work has used neutral infant faces as a control, we have provided secondary analyses in the supplement with LPP to neutral infant faces as the control. Split-half reliability ranged from acceptable to good in this sample (happy: *r*_*SB*_ =.85, neutral *r*_*SB*_ =.77, distressed *r*_*SB*_ =.84, shapes *r*_*SB*_ =.71). Of the 117 participants, 114 participants (97.4%) completed the EEG session. Thirteen additional participants (11.1%) were excluded from EEG analyses due to technical or data quality issues, including missing event markers (*n* = 2; 1.8%), excessive noise in reference channels (*n* = 9; 7.9%), or excessive noise or flat signal in LPP pooling channels (*n* = 2; 1.8%).

### Data analysis plan

The goal of this study was to examine the relations between facets of mindfulness, neural responses to emotion stimuli, and PPD risk. We first tested direct associations between facets of mindfulness and LPP with a series of correlations. Next, we used hierarchical linear regression models to test whether facets of mindfulness and LPP to emotional infant faces in pregnancy predicted postpartum depressive symptoms (accounting for pregnancy symptoms and electrode density) both as main and interaction effects.

#### Data missingness

Missing data from participants who did not complete the EEG (*n* = 3), excluded for noisy EEG data (*n* = 13), or lost to follow up (*n* = 23) were addressed by full information maximum likelihood (FIML) using the *lavaan* and *semTools* packages in *R* (Jorgensen et al., 2022; Rosseel, 2012). A series of *t* and chi-squared tests were used to identify demographic or study variables that predicted missingness. White racial identity was associated with less missing EEG data (χ^2^ = 12.09, *p* <.001). Older age was associated with less missing postpartum data, *F*(1, 115) = 9.58, *p* =.002. Black and/or African American racial identity was associated with more missingness in both EEG data (χ^2^ = 27.00, *p* <.001), and the postpartum assessment (χ^2^ = 5.80, *p* =.016). White and Black racial identity and participant age were used as auxiliary variables via saturated correlations (Graham, 2003) to optimize FIML in all regression analyses presented below.

All continuous predictors were grand-mean centered prior to analysis. Electrode density (16 vs. 32 channels) and depressive symptoms in pregnancy were included as covariates in the first step of all regression models to control for differing electrode montages and baseline levels of depression, respectively. In the second step, all five mindfulness facets along with one of the two LPP variables were added. In the third and final step, interactions between the mindfulness facets and the LPP variable were added. Separate models were conducted for LPP to happy and LPP to distressed infant faces to address the high collinearity between these variables (*r* =.80, variance inflation factor >10). All analyses were reconducted using sensitivity analyses with ocular correction strategy (EOG electrodes or Fp1/FT9/FT10) included as a covariate. Ocular correction strategy was not a significant predictor of PPD (*p* values >.1), and results remained the same, so ocular correction strategy was not included as a covariate in presented models. All analyses were conducted in R (Version 2022.07.2; R Core Team, 2021).

## Results

### Linear relationships between variables

A series of Pearson’s correlations were conducted to assess bivariate correlations between study variables (Table 1). Generally, facets of mindfulness were positively related to each other with small to medium effect sizes (*r* values =.23–.56). Acting with awareness, nonjudgment, and describing facets were inversely related to pregnancy and postpartum depressive symptoms (*p* values <.05); however, nonreactivity was inversely related to depressive symptoms during pregnancy only, and observing was not related to depressive symptoms at either timepoint. Interestingly, LPP to emotional infant faces was not related to mindfulness facets or depressive symptoms (*p* values >.05).

**Table 1:** Descriptive statistics and bivariate correlations between study variables

Variable	*M*	*SD*	1	2	3	4	5	6	7	8
1. FFMQ Observing	26.55	5.32								
2. FFMQ Acting with Awareness	28.23	6.18	.07							
3. FFMQ Nonjudgment	29.37	6.90	.00	.46**						
4. FFMQ Describing	29.50	6.21	.23*	.56**	.46**					
5. FFMQ Nonreactivity	22.70	4.06	.31**	.24*	.33**	.37**				
6. LPP to distressed faces	0.00	3.61	−.14	−.02	−.02	−.01	−.03			
7. LPP to happy faces	0.00	3.86	−.06	.03	.02	.07	.00	.80**		
8. Pregnancy depressive symptoms	36.47	11.04	.02	−.55**	−.53**	−.38**	−.38**	.02	.04	
9. Postpartum depressive symptoms	36.59	8.64	.00	−.48**	−.37**	−.23*	−.04	.09	−.03	.57**

*Note. M* and *SD* are used to represent mean and standard deviation, respectively. FFMQ = Five Facet Mindfulness Questionnaire. LPP = late positive potential residuals adjusting for responses to shapes. Change in Depression = Pregnancy depression symptoms minus postpartum depression symptoms. Table created using apaTables in RStudio (Stanley, 2021). *p <.05. ** *p* <.01

### LPP to emotional infant face regression models

In Step 1, electrode density and pregnancy depressive symptoms were included as covariates to control for differing electrode montages (β = −.011, *p* =.885, 95% CI [−.166,.143]) and baseline levels of depression (β =.655, *p* <.001, 95% CI [.523,.787]), respectively (*R*^2^ =.430) to predict postpartum depressive symptoms. In the second step, all five mindfulness facets along with LPP to happy (see Table 2) or distressed faces (see Table 3) were added. All models accounted for more variance in PPD than Step 1 (Δ*R*^2^ =.001–.037) with the highest variance accounted for by the acting with awareness and LPP to distressed (Δ*R*^2^ =.037) and happy (Δ*R*^2^ =.034) faces. In the third and final step, interactions between the mindfulness facets and the LPP variable were added (see Tables 2 and 3).

**Table 2:** Mindfulness facets and LPP to happy infant faces as predictors of postpartum depressive symptoms

	Regression Statistics					
Postpartum Depressive Symptoms ~ Model Predictors	Main Effects			Interaction Model		
	*R*^*2*^	*β* (SE)	*p*	*R*^*2*^	*β* (SE)	*p*
1. Pregnancy depressive symptoms		**.664 (.065)**	**<.001**		.**663 (.065)**	**<.001**
Electrodes		−.009 (.077)	.910		−.010 (.078)	.898
Observing	.452	.090 (.078)	.252	.452	.087 (.081)	.279
LPP		.010 (.078)	.901		.010 (.079)	.897
Observing × LPP		−−	−−		.010 (.081)	.905
2. Pregnancy depressive symptoms		**.501 (.098)**	**<.001**		**.501 (.098)**	**<.001**
Electrodes		−.026 (.077)	.734		−.016 (.075)	.835
Acting with Awareness	.464	**−.260 (.099)**	**.008**	.491	**−.288 (.098)**	**.003**
LPP		.033 (.079)	.672		.051 (.077)	.507
AWA × LPP		−−	−−		**.217 (.088)**	**.014**
3. Pregnancy depressive symptoms		**.579 (.086)**	**<.001**		**.577 (.086)**	**<.001**
Electrodes		−.013 (.078)	.866		−.011 (.078)	.884
Nonjudgment	.449	−.147 (.091)	.104	.453	−.154 (.092)	.094
LPP		.013 (.078)	.869		.012 (.078)	.875
Nonjudgment × LPP		−−	−−		−.034 (.088)	.703
4. Pregnancy depressive symptoms		**.629 (.075)**	**<.001**		**.628 (.076)**	**<.001**
Electrodes		−.012 (.078)	.874		−.017 (.079)	.834
Describing	.436	−.070 (.083)	.400	.434	−.069 (.083)	.411
LPP		.008 (.079)	.920		.012 (.080)	.881
Describing × LPP		−−	−−		.051 (.086)	.554
5. Pregnancy depressive symptoms		**.696 (.071)**	**<.001**		**.699 (.072)**	**<.001**
Electrodes		.001 (.079)	.986		.003 (.080)	.972
Nonreactivity	.431	.132 (.090)	.143	.434	.136 (.091)	.136
LPP		−.009 (.080)	.911		−.009 (.080)	.907
Nonreactivity × LPP		−−	−−		−.021 (.090)	.812

*Note.* A series of regression models were conducted to assess predictors of postpartum depressive symptoms. Regression statistics are provided. Missing data were handled using full information maximum likelihood, with participant age and White and Black racial identity serving as auxiliary variables. FFMQ = Five Facet Mindfulness Questionnaire. AWA = Acting with Awareness subscale of the FFMQ. LPP = late positive potential. Bold indicates *p* <.05

**Table 3:** Mindfulness facets and LPP to distressed infant faces as predictors of postpartum depressive symptoms

	Regression Statistics					
Postpartum Depressive Symptoms ~ Model Predictors	Main Effects			Interaction Model		
	*R*^*2*^	*β* (SE)	*p*	*R*^*2*^	*β* (SE)	*p*
1. Pregnancy depressive symptoms		**.662 (.065)**	**<.001**		**.656 (.067)**	**<.001**
Electrodes		−.009 (.077)	.905		−.001 (.079)	.992
Observing	.455	.099 (.078)	.205	.454	.105 (.079)	.186
LPP		.078 (.080)	.331		.076 (.081)	.346
Observing × LPP		−−	−−		−.046 (.083)	.577
2. Pregnancy depressive symptoms		**.495 (.099)**	**<.001**		**.490 (.100)**	**<.001**
Electrodes		−.025 (.077)	.741		−.023 (.076)	.760
Acting with Awareness	.467	**−.263 (.098)**	.**007**	.477	**−.266 (.097)**	**.006**
LPP		.084 (.080)	.296		.097 (.083)	.241
AWA × LPP		−−	−−		.050 (.092)	.587
3. Pregnancy depressive symptoms		**.577 (.086)**	**<.001**		**.578 (.086)**	**<.001**
Electrodes		−.013 (.078)	.867		−.016 (.078)	.840
Nonjudgment	.451	−.146 (.090)	.107	.453	−.144 (.090)	.110
LPP		.068 (.080)	.398		.075 (.081)	.360
Nonjudgment × LPP		−−	−−		.035 (.085)	.682
4. Pregnancy depressive symptoms		**.627 (.076)**	**<.001**		**.629 (.076)**	**<.001**
Electrodes		−.013 (.078)	.870		−.011 (.078)	.886
Describing	.438	−.070 (.082)	.394	.437	−.070 (.083)	.395
LPP		.070 (.081)	.383		.063 (.084)	.449
Describing × LPP		−−	−−		−.025 (.083)	.760
5. Pregnancy depressive symptoms		**.693 (.072)**	**<.001**		**.706 (.073)**	**<.001**
Electrodes		.000 (.079)	.999		.006 (.080)	.942
Nonreactivity	.433	.130 (.089)	.145	.435	.140 (.091)	.121
LPP		.070 (.082)	.393		.061 (.082)	.458
Nonreactivity × LPP		−−	−−		−.068 (.102)	.504

*Note.* A series of regression models were conducted to assess predictors of postpartum depressive symptoms. Regression statistics are provided. Missing data were handled using full information maximum likelihood, with participant age and White and Black racial identity serving as auxiliary variables. FFMQ = Five Facet Mindfulness Questionnaire. AWA = Acting with Awareness subscale of the FFMQ. LPP = late positive potential. Bold indicates *p* <.05

### Moderating role of LPP

The addition of the interaction between mindfulness facet and LPP in Step 3 accounted for additional variance in PPD (Δ*R*^2^ =.00–.027) beyond the main effects in Step 2 (see Tables 2 and 3). The interaction between acting with awareness and LPP to happy faces (β =.217, *p* =.014, 95% CI [.045,.390]) accounted for the largest change in variance from Step 2 to Step 3 (Δ*R*^2^ =.027).

**Fig. 3:**
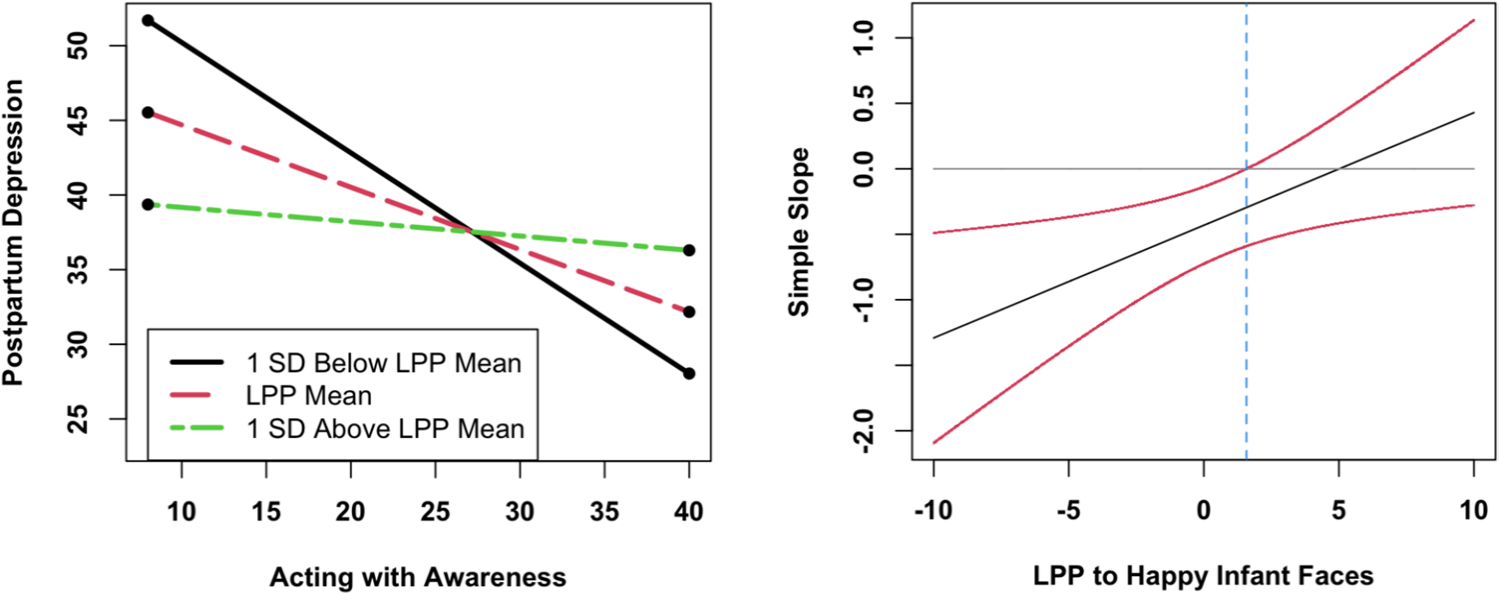
Simple slopes analysis (left) showed that LPP to happy infant faces was a significant predictor at and below the mean for LPP to happy faces. Johnson–Neyman plot (right) shows the region of significance where LPP to happy infant faces was a significant moderator for the relationship between acting with awareness and PPD below 1.58 μV. (Color figure online)

Simple slopes analysis revealed that low acting with awareness was associated with greater postpartum depressive symptoms when LPP to happy infant faces was one standard deviation below (β = −.548, *SE* =.150, *p* <.001) and at the mean (β = −.309, *SE* =.106, *p* =.004; see Fig. 3). Specifically, low acting with awareness in pregnancy was associated with greater postpartum depressive symptoms when LPP was lower than 1.58 μV (see Fig. 3). LPP did not significantly interact with any other mindfulness facets. Of note, no interaction terms survive Benjamini–Hochberg corrections for multiple comparisons (*p* values >.05)

## Discussion

This longitudinal study of 117 women, beginning in midpregnancy and following through 9 weeks postpartum, examined how facets of trait mindfulness and the late positive potential ERP to emotional infant faces in pregnancy interacted to predict PPD. Acting with awareness, nonjudgment, describing, and nonreactivity facets were significantly, negatively correlated with depressive symptoms during pregnancy, but only acting with awareness, nonjudgment, and describing facets were significantly correlated with PPD such that high subscale endorsement during the second trimester of pregnancy was associated with lower PPD. Interestingly, LPP was not significantly associated with either depressive symptoms during pregnancy or postpartum. Importantly, acting with awareness and LPP to happy infant faces interacted to predict PPD such that lower acting with awareness was associated with greater postpartum depressive symptoms for pregnant women with low and average LPP to happy infant faces (adjusting for responses to nonsocial stimuli), but not those with a relatively large LPP to happy faces. This effect was specific to the *acting with awareness* subscale of mindfulness and neural responses to *happy* infant faces, though this effect did not survive correction for multiple comparisons. Such results suggest that an enhanced LPP to positively valenced stimuli may be protective against depression for those low in trait mindfulness.

Negative relationships between acting with awareness and nonjudgment subscales with PPD have been previously identified (Hulsbosch et al., 2022, 2023), such that higher levels of trait mindfulness subscales were associated with lower levels of postpartum depressive symptoms. While Hulsbosch and colleagues (2023) found that nonjudgment appeared to have a larger protective factor than acting with awareness, in the present study, only acting with awareness remained a significant predictor of depression after controlling for baseline depressive symptoms and LPP to emotional stimuli. Acting with awareness is defined as actively paying attention to the current moment (Baer et al., 2006) and has previously been highlighted as the only subscale both inversely associated with depression and rumination and positively associated with reappraisal strategies during emotion regulation (Shallcross et al., 2020), suggesting that the acting with awareness subscale may play a unique role in promoting generally adaptive emotion regulation strategies.

Relatedly, acting with awareness interacted with LPP to happy faces, but not distressed faces. Alterations in responses to both negative and positively valenced stimuli have been implicated as risk factors for depression (Hill et al., 2023; Medeiros et al., 2020); however, alterations in responses to negative-valenced stimuli appear to be general risk factors for psychopathology more broadly with mixed effects for depression risk (Kujawa & Burkhouse, 2017; Nikolin et al., 2022). In contrast, emerging evidence has suggested a specific role of responses to positively valence system alterations as risk factors for depression (Keren et al., 2018). While acting with awareness promotes opportunities to notice and engage with positive stimuli to promote mental well-being, our findings suggest that heightened neural reactivity to positive stimuli may further promote postpartum mental health, especially for individuals with a lower tendency to be present-minded (i.e., low acting with awareness) by increasing neural engagement with positive stimuli. This relationship may be even more pronounced for new mothers interacting with happy infant face cues in their postpartum environment with potential impact to maternal bonding with her infant. Indeed, women with heightened acting with awareness are less likely to report difficulties feeling connected to their infant (Garon-Bissonnette et al., 2024).

Interestingly, LPP was not associated with depressive symptoms during pregnancy or in postpartum in contrast to previous work which has shown that blunted LPP to distressed infant faces (Rutherford et al., 2016) and increased LPP to happy infant faces (Mulligan et al., 2022) was associated with increased postpartum depressive symptoms. This is somewhat surprising given the large amount of evidence supporting a blunted LPP to emotional stimuli for individuals with elevated depressive symptoms (for a review, see Hajcak & Foti, 2020; Proudfit et al., 2015); however, main effects of LPP tend to have small effect sizes (Mulligan et al., 2022; Proudfit et al., 2015) and may be more apparent in larger samples. Additionally, it is important to note that our study is unique in that we tested predictors of depressive symptoms longitudinally, while previous work has modeled the relationships between LPP and depression concurrently (Rutherford et al., 2016). Further, differences in both task and preprocessing steps may also account for discrepancies in findings. For example, a recent study using multiple preprocessing pipelines identified in previous studies to extract LPP found mixed results for group differences in LPP for individuals with and without depression (Nikolin et al., 2022). Such findings highlight the importance of standardizing preprocessing steps and task parameters across studies for appropriate comparison of findings.

Importantly, the results from the present analyses must be held in consideration of study limitations. First, emotional stimuli used in this study were limited to infant faces which may result in different responses than general emotional stimuli or even adult faces as used in other depression studies outside of pregnancy. Within pregnancy, LPP to emotional infant faces were found to have different associations with reflective functioning three months postpartum that were not shown for LPP to emotional adult faces (Rutherford et al., 2018). An important next step will be to understand how mindfulness and LPP to emotional stimuli interact to predict depression outside of pregnancy and with generalized emotional content rather than content specific (i.e., infant faces) stimuli. Additionally, most of the sample was below the IDAS clinical screening cut off for major depressive disorders which may partially explain why a linear relationship between LPP and depression scores was not replicated in these data. It may also be the case that a general depression scale like the IDAS used in this study may be limited in measuring peripartum depression specifically than peripartum assessment questionnaires. Future studies should replicate these results in studies oversampled for clinical depression with use of questionnaires designed for assessing PPD rather than depression more broadly. Finally, examination of all five mindfulness facets was exploratory and resulted in testing multiple models revealing an interaction between LPP to happy infant faces and acting with awareness that did not survive correction for multiple comparisons, so replication of these results is necessary and should be interpreted with caution.

## Conclusion

The present study sought to understand how neural emotional processing and facets of trait mindfulness interact as predictors of PPD. Pregnant women completed an infant face matching task while continuous EEG was recorded to index neural emotional processing. Acting with awareness and LPP to happy infant faces interacted to predict postpartum depressive symptoms after controlling for pregnancy depressive symptoms. Importantly, these results highlight the importance of considering neural processes such as emotional processing that may impact the known link between mindfulness and depression and the importance of studying trait mindfulness as a set of facets rather than a general construct. Further, the unique interaction between acting with awareness and LPP to happy, but not distressed, faces underscores the importance of positive valence system functions in pathways to depression risk. Finally, such results promote the utility of EEG research across peripartum to inform the understanding of depression risk and intervention targets.

## Supplementary Information

Below is the link to the electronic supplementary material.

Supplementary file1 (DOCX 20.1 KB)

## Data Availability

Data and materials are available upon request to the corresponding author. This experiment was not preregistered.

## Code availability

All code for data collection and analysis is available upon request to the corresponding author.
