# Supplementary material for: Neural reactivity to infant faces and trait mindfulness as prospective predictors of postpartum depressive symptoms
Source: Cogn Affect Behav Neurosci. 2025 Jun 20;26(1):294–305. doi: 10.3758/s13415-025-01319-8 (PMC12232934; doi:10.3758/s13415-025-01319-8)
Supplement: Supplementary file 1 — Supplementary file1 (DOCX 20.1 KB) [file 13415_2025_1319_MOESM1_ESM.docx]

**Supplemental**

Provided that previous work has residualized emotional infant faces to neutral infant faces rather than shapes, we have provided supplemental analyses with LPP residualized to neutral faces (Table A).

*Table A. Mindfulness facets and LPP (residualized to neutral infant faces) to emotional infant faces as predictors of postpartum depressive symptoms*

|  | Regression Statistics | | | | | | | | | | | | |  |
| --- | --- | --- | --- | --- | --- | --- | --- | --- | --- | --- | --- | --- | --- | --- |
| Postpartum Depressive Symptoms ~ |  | Happy Ixn Models | | | |  | |  | Distress Ixn Models | | | |  | |
| Model Predictors | *R^2^* | | | *β (SE)* | *p* | | *R^2^* | | | *β (SE)* | | *p* | |  |
| 1. Pregnancy depressive symptoms  Electrodes  Observing  LPP  Observing x LPP | .456 | | **.658 (.066)**  -.009 (.078)  .086 (.078)  -.007 (.082)  .070 (.078) | | **<.001**  .906  .270  .937  .368 | | .455 | | | **.670 (.065)**  -.001 (.079)  .099 (.079)  .039 (.086)  .037 (.080) | **<.001**  .987  .209  .646  .640 | | |  |
| 2. Pregnancy depressive symptoms  Electrodes  Acting with Awareness  LPP  AWA x LPP | .485 | | **.509 (.098)**  -.003 (.077)  **-.271 (.097)**  -.010 (.078)  **.202 (.086)** | | **<.001**  .968  **.005**  .895  **.018** | | .458 | | | **.505 (.100)**  -.024 (.079)  **-.254 (.098)**  .004 (.085)  -.012 (.110) | **<.001**  .765  **.010**  .964  .912 | | |  |
| 3. Pregnancy depressive symptoms  Electrodes  Non-judgment  LPP  Non-judgment x LPP | .447 | | **.577 (.087)**  -.017 (.079)  -.146 (.092)  -.026 (.081)  -.007 (.092) | | **<.001**  .835  .113  .749  .937 | | .451 | | | **.581 (.086)**  -.004 (.080)  -.146 (.090)  .019 (.086)  .044 (.093) | **<.001**  .956  .106  .826  .634 | | |  |
| 4. Pregnancy depressive symptoms  Electrodes  Describing  LPP  Describing x LPP | .435 | | **.634 (.078)**  -.003 (.079)  -.040 (.084)  -.025 (.080)  .145 (.083) | | **<.001**  .972  .637  .752  .080 | | .440 | | | **.632 (.076)**  -.002 (.080)  -.059 (.083)  .020 (.087)  .054 (.086) | **<.001**  .983  .481  .816  .528 | | |  |
| 5. Pregnancy depressive symptoms  Electrodes  Non-reactivity  LPP  Non-reactivity x LPP | .424 | | **.686 (.074)**  .000 (.080)  .135 (.090)  -.049 (.082)  .053 (.089) | | **<.001**  .996  .135  .551  .549 | | .434 | | | **.697 (.072)**  .009 (.081)  .132 (.089)  .032 (.088)  .020 (.096) | **<.001**  .909  .139  .713  .837 | | |  |

*Note.* A series of regression models were conducted to assess predictors of postpartum depression. Regression statistics are provided. Missing data were handled using Full Information Maximum Likelihood, with participant age and White and Black racial identity serving as auxiliary variables. Ixn = Interaction. FFMQ = Five Facet Mindfulness Questionnaire. AWA = Acting with Awareness subscale of the FFMQ. LPP = Late Positive Potential. Bold indicates *p* < .05.
